# Assessment of training-associated changes of the lumbar back muscle using a multiparametric MRI protocol

**DOI:** 10.3389/fphys.2024.1408244

**Published:** 2024-10-17

**Authors:** Marta B. Maggioni, Renat Sibgatulin, Martin Krämer, Daniel Güllmar, Jürgen R. Reichenbach

**Affiliations:** ^1^ Medical Physics Group, Institute for Diagnostic and Interventional Radiology, University Hospital Jena–Friedrich Schiller University Jena, Jena, Germany; ^2^ Department of Biomedical Engineering, University of Basel, Basel, Switzerland

**Keywords:** qMRI, training, lumbar back muscle, diffusion, IVIM

## Abstract

Adaptations in muscle physiology due to long-term physical training have been monitored using various methods: ranging from invasive techniques, such as biopsy, to less invasive approaches, such as electromyography (EMG), to various quantitative magnetic resonance imaging (qMRI) parameters. Typically, these latter parameters are assessed immediately after exercise. In contrast, this work assesses such adaptations in a set of qMRI parameters obtained at rest in the lumbar spine muscles of volunteers. To this end, we developed a multiparametric measurement protocol to extract quantitative values of (water) T_2_, fat fraction, T_1_, and Intra Voxel Incoherent Motion (IVIM) diffusion parameters in the lumbar back muscle. The protocol was applied to 31 healthy subjects divided into three differently trained cohorts: two groups of athletes (endurance athletes and powerlifters) and a control group with a sedentary lifestyle. Significant differences in muscle water T_2_, fat fraction, and pseudo-diffusion coefficient linked to microcirculatory blood flow in muscle tissue were found between the trained and untrained cohorts. At the same time, diffusion coefficients (resolved along different directions) provided additional differentiation between the two groups of athletes. Specifically, the strength-trained athletes showed lower axial and higher radial diffusion components compared to the endurance-trained cohort, which may indicate muscle hypertrophy. In conclusion, utilizing multiparametric information revealed new insights into the potential of quantitative MR parameters to detect and quantify long-term effects associated with training in differently trained cohorts, even at rest.

## 1 Introduction

Physical activity and muscle training have several positive effects, such as improving cardiovascular health (Pinckard et al., 2019), increasing muscle resistance to fatigue (Nystoriak and Bhatnagar, 2018), and reducing the risk of developing diseases such as type II diabetes (Neufer et al., 2015) or colorectal cancer (Samad et al., 2005). The fact that consistent physical activity modifies skeletal muscle morphology is widely accepted (Flück et al., 2019), but assessing and quantifying these modifications usually requires invasive techniques such as muscle biopsies. Biopsy-based studies have demonstrated significant adaptations of muscle tissue to long-term physical activity, including changes in muscle fiber type composition and size (Andersen and Aagaard, 2010; Meijer et al., 2015). They have also successfully shown distinct effects of different training regimens on metabolism- and contraction-related cellular parameters (Terzis et al., 2010; Flück et al., 2019).

A prominent non-invasive tool for studying the activation of muscle tissue is electromyography (EMG). EMG records the electrical activity that leads to muscle contraction (Mills, 2005) and has been shown to aid in both diagnosis and the study of muscle function, although caution should be exercised in interpreting it in terms of muscle force or timing of contraction (Roberts and Gabaldón, 2008). A recent surface electromyography study observed a significant difference in muscle activation in subjects with different exercise routines (Schönau and Anders, 2023). In this study, both endurance athletes and controls performed better than strength athletes in a 10-minute back muscle endurance test at a load of 50% of upper body weight.

Possible long-term effects associated with regular training, detectable by EMG or biopsy, are less well-researched with quantitative magnetic resonance imaging (qMRI). There are only a few MRI-based studies on this topic (Le Rumeur et al., 1994; Sun et al., 2018; Berry et al., 2020; Marth et al., 2022), each generally focusing on only a few parameters, but highlighting the possible impact of exercise-induced muscle damage, affecting the transverse relaxation time T_2_ and significant changes in the fat fraction between the trained and untrained cohorts (Emanuelsson et al., 2022).

T_2_ relaxation time is a well-established parameter in MRI, frequently used for evaluating muscle tissues. It is important to distinguish water (muscle) T_2_ and global T_2._ Global T_2_ includes signals from both muscle and fat without differentiation. Water T_2_ has been shown to increase after exercise, and the origin of this phenomenon has been linked to an accumulation of osmolytes due to increased fluid influx (Patten et al., 2003; Cagnie et al., 2011). Apparent diffusion coefficients (ADC) have also been shown to increase after exercise (Hiepe et al., 2013; Kolmer et al., 2023), which is thought to be due to increased fluid in muscles during and after a strenuous exercise session. Other studies using Intravoxel Incoherent Motion (IVIM) MRI sequences have also demonstrated increased microcirculatory blood flow after exercise (Nguyen et al., 2016; Federau et al., 2020; Englund et al., 2022).

Two other MRI parameters that have been used to characterize muscle tissue morphology are fat fraction and T_1_ relaxation time. Increased fat fraction has been associated with reduced power output per muscle unit and with low back pain (Hildebrandt et al., 2017; Schlaeger et al., 2019). The level of fat replacement of muscle tissue can be quantitatively assessed by MRI, exploiting the chemical shift between the proton signals of fat and water. Furthermore, changes in T_1_ immediately after exercise have been associated with increased blood perfusion and tissue temperature (Fleckenstein et al., 1988; de Sousa et al., 2011), but the origin of this phenomenon is not fully understood.

Most of the qMRI literature on the effect of exercise on skeletal muscle has focused on measuring changes in some quantitative MRI parameters, such as T_2_ and diffusion, immediately after a strenuous exercise session (Cagnie et al., 2011; Hiepe et al., 2013). However, long-term effects associated with regular physical activity on qMRI parameters appear to be relatively understudied (Berry et al., 2020; Keller et al., 2020).

To bridge this gap, we developed a multi-parametric protocol to comprehensively characterize back muscle morphology and to investigate whether long-term physical activity and type of training are associated and detectable in changes of qMRI parameters. This protocol included well-established parameters, such as muscle water T_2_ and fat fraction, which have been used before to characterize skeletal muscle composition (even at rest), and less well-studied parameters such as T_1_, diffusion (Damon et al., 2002; Sinha et al., 2006; Karampinos et al., 2010) and perfusion-related parameters (Filli et al., 2015). Diffusion parameters have been shown to be sensitive to microstructural changes such as muscle fiber dispersion, organisation and size which might aid in the differentiation of the impact of different training regimes on the skeletal muscle tissue. Additionally, physical activity is associated with vascular adaptation which might influence perfusion values even at rest (Green et al., 2012; Welsch et al., 2013).

The protocol was applied to the muscles of the lumbar spine (specifically the area between L4 and L5) rather than the more commonly investigated skeletal muscles of the calf and thigh. This choice was made because the lumbar paraspinal muscles are increasingly being linked to nonspecific low back pain, which is one of the most common causes of disability worldwide (Balagué et al., 2012). The exact causes behind this condition are not yet fully understood and there is no consensus on the precise role of paraspinal muscles on nonspecific low back pain (Ranger et al., 2017; Hodges et al., 2021). This may be due to the fact that traditional clinical MRI examinations only allow qualitative morphological evaluations, while quantitative results have the potential to assess the underlying tissue function (Sollmann et al., 2022). Furthermore, physical activity has been shown to be a viable treatment, but there is still debate about which type of physical activity is most beneficial (Shipton, 2018). Quantitatively evaluating the long-term effects associated with training in healthy cohorts and at rest using the proposed protocol may pave the way for treatment monitoring in low back pain.

In this study, we hypothesize that while parameters like water T_2_ and fat fraction can distinguish between trained and untrained groups even at rest, a deeper analysis involving diffusion and perfusion coefficients is necessary to discern the impacts of various training regimens on both the skeletal muscle composition and structure.

## 2 Materials and methods

### 2.1 Participants

Three cohorts of male subjects were studied: Endurance athletes (long-distance runners and cyclists, n = 10), strength athletes (powerlifters, n = 11), and a control group (n = 10) with a non-athletic lifestyle. All subjects were between 20 and 31 years of age, average age and BMI per cohort are detailed in Table 1. The institutional ethics committee approved the study. All participants provided written informed consent. Athletes were recruited through advertisements in local sports clubs and gyms. They were enrolled into the study if they trained at least four times per week (training session duration of at least 1 h) and had been training for at least the previous 3 years. The sedentary control group, recruited from the local university student population through advertisements placed in university canteens, was required to have no regular participation in physical activities such as regular sport or any kind of deliberate physical training and a mostly sedentary occupation without intense physical activity. Adherence to these criteria, as well as the absence of back pain in all participants, was self-reported. Subjects underwent MRI examinations in supine position with the vendor’s spine coil in a 3T Magnetom Prisma fit scanner (Siemens Healthineers, Erlangen, Germany).

**TABLE 1:** Summary of the ages and BMI of the analyzed volunteers. Values are given as mean ± standard deviation for each cohort. The ages across the groups showed no significant statistical differences, while the BMI of the strength training cohort was significantly different from the other two cohorts.

Cohort	Age [years]	BMI [kg/m^2^]
Control	29 ± 3	23 ± 2
Endurance	25 ± 3	21 ± 1
Strength	26 ± 2	28 ± 2

### 2.2 MRI protocol

The MRI protocol included sequences to determine relaxation parameters (i.e., water T_2_ and T_1_), fat fraction, and IVIM parameter values. Quantitative water T_2_ values were determined using a turbo spin echo (TSE) sequence with 28 echo times ranging from 7 ms to 218 ms, while the T_1_ relaxation time constants were determined using a variable flip angle (VFA) gradient echo sequence (Christensen et al., 1974) with five different flip angles (5°, 11°, 18°, 24°, 32°). For accurate VFA-based T_1_ determination, especially at higher field strengths and in areas where the RF signal is attenuated (e.g., lower lumbar and abdominal regions), correction of the B_1_-field is essential, which was performed here by acquiring an additional B_1_ calibration scan using the Bloch-Siegert method (Sacolick et al., 2010). The in-plane spatial resolution was (1.5 × 1.5) mm^2^ for all sequences, and the slice thickness was 3 mm (except for the TSE sequence, which used a slice thickness of 6 mm).

Diffusion-weighted images (DWI) were acquired with a multi-slice spin-echo EPI (echo planar imaging) sequence with TR/TE = 1,600/63 ms using four averages and three orthogonal directions of diffusion encoding aligned with the right-handed RAS system (each direction was repeated twice with opposite polarities). For each direction, the following 19 b-values were used: 0, 5, 15, 20, 30, 40, 45, 55, 60, 70, 75, 90, 105, 120, 135, 150, 300, 450, 600 s/mm^2^, with particular emphasis placed on acquiring low b-values as they have been shown to aid in IVIM based quantification (Ye et al., 2019). The resolution for the DWI data was (2.3 × 2.3 × 8) mm^3^. Spectral Attenuated Inversion Recovery (SPAIR) fat saturation was used.

All images (from L4 to L5) were acquired in axial orientation. The muscles included in the field of view were multifidus (MF), erector spinae (ES), psoas major, and quadratus lumborum. However, the latter two muscles could not be evaluated consistently in all subjects due to artifacts caused by respiration and bowel motion and were excluded from the analysis (cf. section 1.3 of the Supplementary Material). The total acquisition time was 43 min. All relevant acquisition parameters can be found in Table 2. MRI scans were performed at rest, at least 48 h after the last exercise session, to exclude exercise-related parameter changes.

**TABLE 2:** Parameters of the MRI protocol. TR stands for repetition time, TE for echo time and TA for acquisition time. VFA GRE is a variable flip angle gradient echo sequence, TSE for turbo spin echo and finally EPI for echo planar imaging.

	VFA GRE	B_1_ map	TSE	Diffusion EPI
TR [ms]	16	20	5,000	1,600
TE [ms]	2.4	1.76	7.8–218.4	63
Voxel [mm^3^]	1.5 × 1.5 × 3	1.5 × 1.5 × 3	1.5 × 1.5 × 6	2.3 × 2.3 × 8
FOV [mm^3^]	380 × 285 × 3	380 × 285 × 3	380 × 285 × 6	300 × 187 × 8
Averages	-	-	-	4
Saturation bands	-	-	yes	-
Slices	40	40	20	10
TA	15 min	40 s	16 min	12 min

### 2.3 MRI data processing and analysis

Regions of interest (ROIs) were manually drawn to segment the multifidus and erector spinae muscles based on apparent diffusion coefficient (ADC) maps for each subject, avoiding fascia, connective tissue and subcutaneous fat. Since there were no significant differences between the left and right sides in any participants, the voxels from the quantitative maps (created through voxel-wise fitting) were pooled to calculate the reported averages.

The water T_2,_ fat fraction and T_1_ maps were calculated by fitting the expected physical signal model to the measured signals. For T_2_ quantification, extended phase graphs (EPG) (Santini et al., 2021) were used to remove the signal contributions from intra-muscular fat. EPG allows the simulation of a spin system response to an arbitrary MRI sequence and enables the retrieval of accurate T_2_ values in multicompartment voxels containing lipids and muscle tissue (Marty et al., 2016). It is worth noting that the EPG method also returns fat fraction maps, which were used for fat fraction quantification and comparison between the cohorts. For T_1_ quantification, the values of the input flip angles were corrected using the B_1_ maps before fitting (Sacolick et al., 2010) using the code available at MedPhysJena/b1_corrected_t1_vfa.

Preprocessing of the IVIM diffusion data included denoising (using Mrtrix3 (Veraart et al., 2016b; Veraart et al., 2016a; Tournier et al., 2019)), averaging the repetitions, and geometric averaging the opposite polarities of the diffusion gradients to correct cross-terms between the imaging and diffusion gradients (Neeman et al., 1991). No corrections were made for susceptibility-induced distortions or eddy currents.

A scalar IVIM model from Equation 1 was fitted to each gradient encoding direction independently using DIPY (Garyfallidis et al., 2014), see Appendix A1 for details. For the IVIM model, the following signal equation was used

$$S\left(b_{i,j}\right)=S_{0,j}\;\left[\;f_{j}\;\exp\left(-b_{i,j}D_{j}^{*}\right)+\left(1-f_{j}\right)\exp\left(-b_{i,j}D_{j}\right)\;\right],$$ (1)

where *j* indices denote the directions of the applied diffusion gradients (i.e., *x/AP*, *y/LR*, or *z/IS*). $S\left(b_{i,j}\right)$ and $S_{0,j}$ are the signal intensities for the *i*th *b*-value and *b* = 0, respectively, $f_{j}$ is the perfusion fraction, $D_{j}^{*}$ is the scalar pseudo-diffusion coefficient, and $D_{j}$ is the diffusion coefficient, associated with the *j*th gradient encoding direction. In each subject and each ROI, values of the perfusion fraction and the pseudo-diffusion coefficient, obtained from different gradient directions, were not significantly different. Thus, the three corresponding maps of $f_{j}$ , $D_{j}^{*}$ as well as $S_{0,j}$ were averaged, yielding a set of 6 maps per subject: *S*_*0*_ *, f, D* ^*^ *, D*_*x*_ *, D*_*y*_, and *D*_*z*_. Furthermore, *D*_*x*_ and *D*_*y*_ were additionally averaged to form *D*_*xy*_, hereafter referred to as the *radial diffusion coefficient*, while *D*_*z*_ was reported as and referred to as the *axial diffusion coefficient*.

It's important to note that the diffusion coefficients *D*_*z*_ and *D*_*xy*_ do not inherently represent quantitative parameters of the muscle fibers, as radial and axial diffusivities do, and they may be influenced by the muscle fiber orientation. However, neither the full rank-2 diffusion tensor nor its eigenvectors could be reconstructed from the three orthogonal diffusion gradient directions. Yet, we believe that, given the characteristic geometry of the muscle fibers, one of the three gradient directions (specifically z/IS) approximately aligns with the fiber orientation, while the other two are roughly perpendicular. Therefore, we propose considering these diffusion coefficients as a rough approximation of the aforementioned quantitative parameters.

Differences between the averaged values extracted from ROIs in the control, endurance, and strength cohorts for T_1_, water T_2_, fat fraction, and all IVIM parameters, as well as the age and BMI of the subjects, were tested in Python (version 3.12) using a non-parametric Kruskal–Wallis test (Kruskal and Wallis, 1952) part of SciPy (Virtanen et al., 2020) version 1.91. If the Kruskal–Wallis test indicated significance, Dunn’s *post hoc* pairwise comparisons were performed (Dunn, 1961) with scikit-posthocs (Terpilowski, 2019) version 0.8.1. Statistical significance was considered at *p* ≤ 0.05. The *p*-values reported in the results are from Dunn’s *post hoc* comparisons, and all other *p*-values can be found in the Supplementary Material.

## 3 Results

**FIGURE 1:**
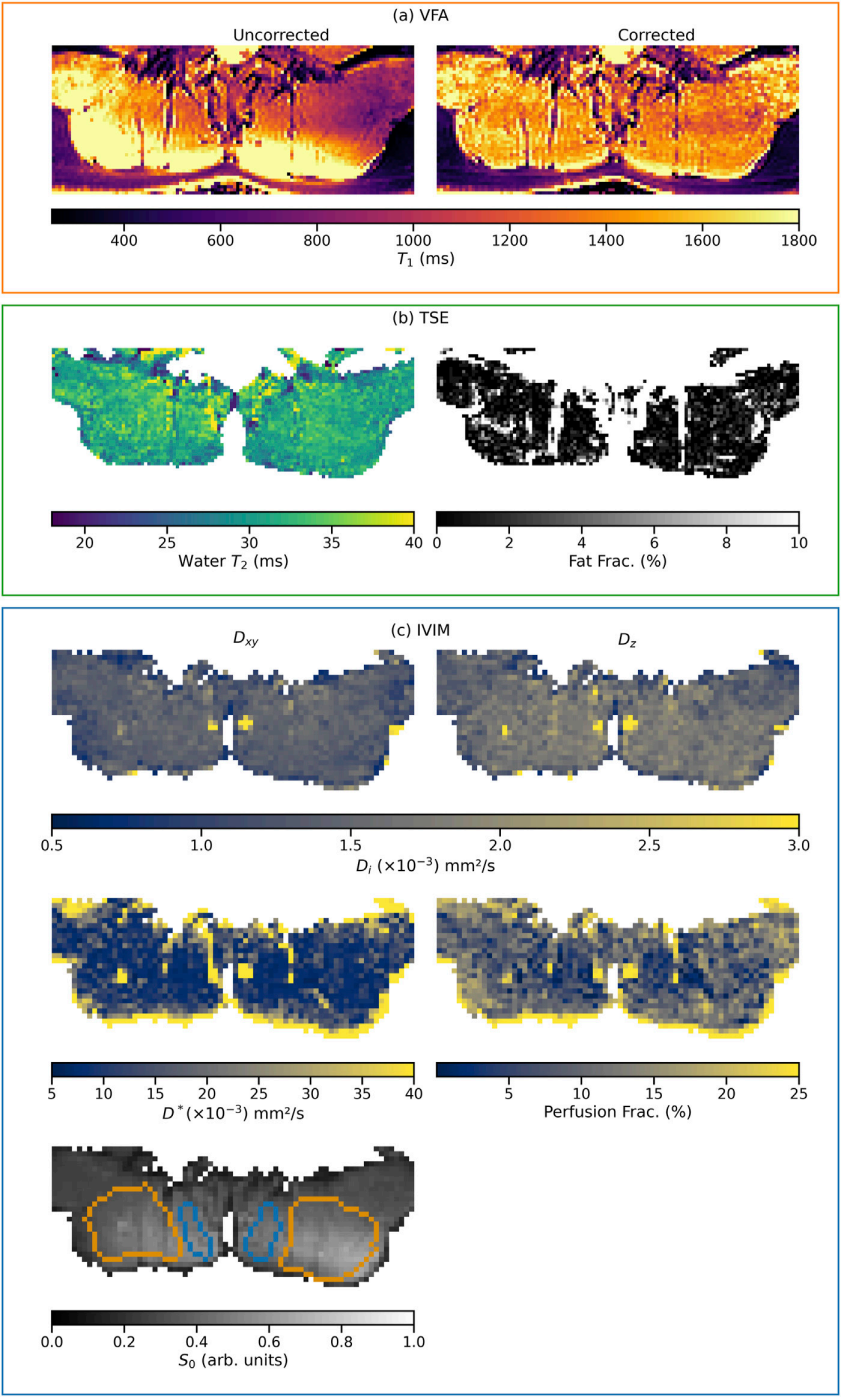
Example parameter maps from one subject of the endurance cohort. The grouping of the maps, indicated by the frame color, represents the acquisition from which they originate (VFA, TSE, or IVIM). Maps sharing a color bar are windowed identically. In the corrected T_1_ relaxation time map, the successful B_1_-field inhomogeneity correction is clearly visible compared to the uncorrected map **(A)**. Section **(B)** shows the water T_2_ and fat fraction maps extracted from the EPG. Section **(C)** shows the maps of the diffusion coefficients $D_{xy}$ , *D*_*z*_, the pseudo-diffusion coefficient *D* ^*^, and the perfusion fraction *f*. Additionally, the ROIs used for quantitative analysis are outlined on top of the map of *S*_*0*_ (multifidus in blue and erector spinae in orange).

Figure 1 shows exemplary parameter maps from a single subject for all analyzed qMRI parameters of the proposed multi-parametric MR protocol, grouped by acquisition sequence (corresponding maps for subjects of the two other cohorts analyzed in this work are available in section 1.4 of the Supplementary Material). Additionally, the ROIs used in this subject are outlined on the *S*_*0*_ image.

**FIGURE 2:**
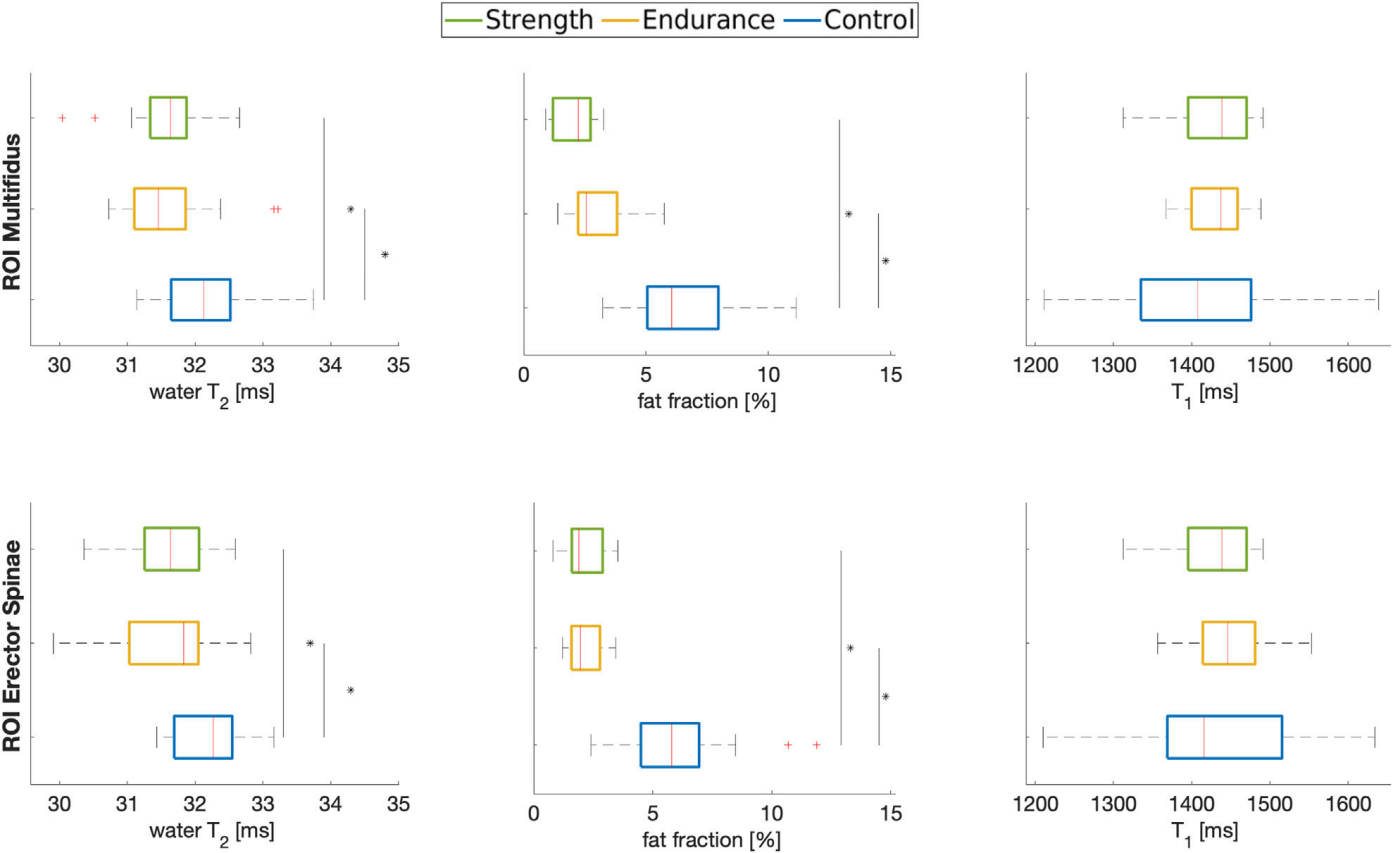
Boxplots of water T_2_, fat fraction and T_1_ in the multifidus (*top row*) and erector spinae (*bottom row*) muscles of the three cohorts analyzed in this work. * indicates significance at *p* < 0.05 between indicated cohorts.

**FIGURE 3:**
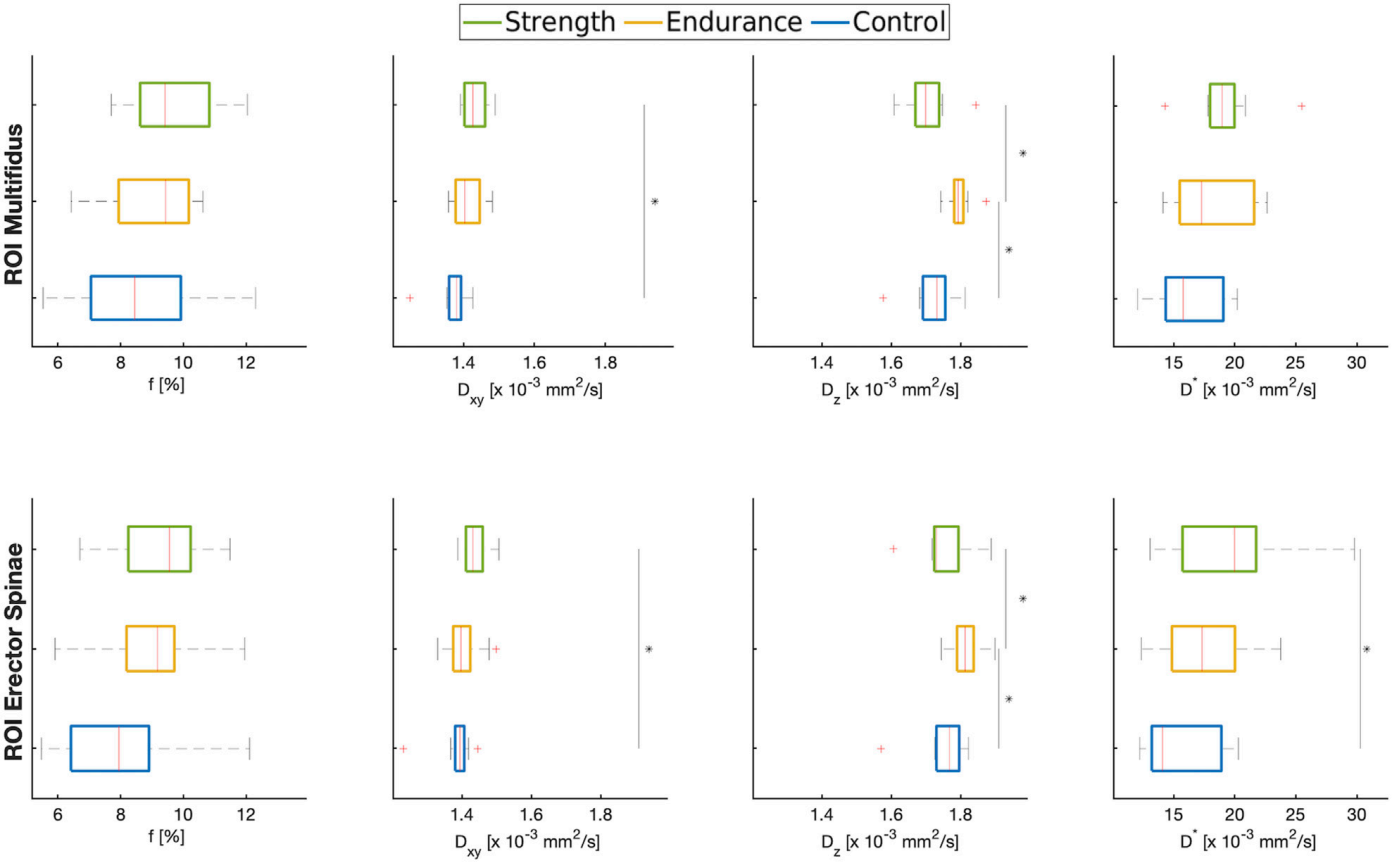
Boxplots of ROI-averaged f, $D_{\text{xy}}$ , *D*_*z*_, and D* in the multifidus (top row) and erector spinae (bottom row) muscles of the three cohorts analyzed in this work. * indicates significance at *p* < 0.05 between indicated cohorts.

Figures 2, 3 summarize the results of the quantitative ROI analyses for both muscles and all cohorts. Specifically, Figure 2 includes the transverse relaxation parameters water T_2_, the fat fraction (both calculated using the EPG approach) and the longitudinal relaxation parameter T_1_. For both multifidus and erector spinae muscles, control subjects had significantly higher water T_2_ values (*p* = 0.012 for the comparison endurance athletes vs. controls for multifidus and *p* = 0.018 for erector spinae; *p* = 0.025 for the comparison strength athletes vs. controls for multifidus and *p* = 0.017 for erector spinae). Both athlete cohorts had significantly lower fat fractions compared to the control group, with the strength cohort showing particularly low fat fractions in the multifidus. However, even after implementing B_1_ correction, which removed large inhomogeneities in the T_1_ map, as shown in the top left section (a) of Figure 1, no significant difference in T_1_ relaxation times was found between the cohorts. The mean values of all quantified parameters for each group are shown in Tables 3, 4. Additionally, fat fraction maps were also obtained calculating the ratio of fat to the combined signal of fat and water in separately acquired clinically available 2-point Dixon sequence (Dixon, 1984). The two methods reveal the same differences between the cohorts (i.e., athletes’ fat fraction is significantly lower). The 2-point Dixon sequence parameters and fat fraction results are shown in Table 1a and 2a of the Supplementary Material (section 1.2).

**TABLE 3:** Summary of the values of water T_2_, fat fraction and T_1_ extracted using the MRI protocol. Values are given as mean ± standard deviation for each cohort. *MF—M. multifidus; ES—M. erector spinae.*

Cohort	Water T_2_ (ms) MF	Water T_2_ (ms) ES	Fat fraction EPG (%) MF	Fat fraction EPG (%) ES	T_1_ (ms) MF	T_1_ (ms) ES
Control	32.1 ± 0.7	32.1 ± 0.5	7 ± 3	6 ± 2	1,400 ± 100	1,400 ± 120
Endurance	31.7 ± 0.7	31.6 ± 0.8	2.9 ± 1.2	2.1 ± 0.7	1,430 ± 40	1,440 ± 60
Strength	31.5 ± 0.6	31.6 ± 0.6	2.0 ± 0.8	2.1 ± 0.9	1,420 ± 50	1,430 ± 70

**TABLE 4:** Summary of the different parameters extracted using the MRI protocol. Values are given as mean ± standard deviation for each cohort. *MF—M. multifidus; ES—M. erector spinae.*

Cohort	$f\;\left(\%\right)$ MF	$f\;\left(\%\right)$ ES	$D_{\text{xy}}$ ( $\times 10^{-3}$ mm/s^2^) MF	$D_{\text{xy}}$ ( $\times 10^{-3}$ mm/s^2^) ES	$D_{z}$ ( $\times 10^{-3}$ mm/s^2^) MF	$D_{z}$ ( $\times 10^{-3}$ mm/s^2^) ES	$D^{*}$ ( $\times 10^{-3}$ mm/s^2^) MF	$D^{*}$ ( $\times 10^{-3}$ mm/s^2^) ES
Control	8 ± 2	7.8 ± 1.9	1.37 ± 0.04	1.38 ± 0.05	1.72 ± 0.06	1.75 ± 0.08	16 ± 3	15 ± 3
Endurance	9.0 ± 1.4	8.9 ± 1.7	1.41 ± 0.04	1.40 ± 0.05	1.79 ± 0.03	1.81 ± 0.04	17 ± 3	17 ± 4
Strength	9.7 ± 1.5	9.5 ± 1.8	1.43 ± 0.03	1.43 ± 0.03	1.70 ± 0.06	1.75 ± 0.07	19 ± 3	20 ± 5

Figure 3 shows the estimated means of *f*, $D_{xy}$ and $D_{z}$ as well as $D^{*}$ .The mean perfusion fraction in both training cohorts (f ≈ 9%) was slightly higher than in controls (f ≈ 7–8%). This trend was present in both muscles, although it wasn’t statistically significant.

The radial diffusion coefficient tends to be higher in the strength training cohort than in the controls and the endurance athletes, with the difference between the strength cohort and the controls being statistically significant (*p* = 0.001 for multifidus and *p* = 0.011 for erector spinae). The axial diffusion coefficient, on the other hand, shows different trends in the two athlete cohorts compared to the controls: the endurance cohort shows a higher value of D_z_ (*p* = 0.001 for multifidus and *p* = 0.026 for erector spinae) compared to the strength cohort and to the control group (*p* = 0.024 and *p* = 0.044 for multifidus and erector spinae, respectively).

The pseudo-diffusion coefficient D^*^ was also higher for the athlete cohorts, with significant differences between the strength cohort and the control group (*p* = 0.02 for erector spinae).

## 4 Discussion

Among the numerous musculoskeletal studies using MRI, only a limited number of similar studies conducted at rest, exists. Still, they have examined only selected parameters at a time, focused only on athlete cohorts without including a control group (Marth et al., 2022), or investigated solely a single trained group (Sun et al., 2018; Berry et al., 2020; Keller et al., 2020) without including a differently trained cohort. Based on the synopsis and analysis of the multiparametric MRI protocol used in this work, our results suggest that although the water T_2_ and fat fraction could distinguish between trained and untrained cohorts, only the addition of the IVIM-based diffusion coefficients helped to reveal differences between the two cohorts of athletes.

The observation of lower water T_2_ values in the athletes is consistent with the findings of Le Rumeur et al. (1994), Sun et al. (2018), but contradicts those of Keller et al. (2020). However, it is important to note that all these studies assessed global T_2_ values. Sun *et al.* hypothesize that the lower T_2_ values in athletes are due to a higher concentration of macromolecules, which increase the bound water component, thus shortening T_2_. This hypothesis might further relate to the diffusion results presented in this work. However, it should be emphasized that all these results were obtained from leg skeletal muscles, which are characterized by a smaller fat fraction (compared to back muscles) and more directly influenced by movements, including walking to the MRI examinations. In contrast, the T_2_ values for the multifidus and erector spinae muscles reported in other studies for healthy controls are typically higher than those measured in this work (D’hooge et al., 2013; Huang et al., 2020). This discrepancy is likely because those studies did not account for the signal contribution from the fat fraction in their measurement of global T_2_ values.

The higher fat fraction observed in the control cohort aligns with the work of Emanuelsson et al. (2022) who evaluated the multifidus and erector spinae as a single ROI. The fat fraction maps were derived as a byproduct of the EPG T_2_ fitting process. Correction for inaccuracies in the slice profile (a well-known source of errors in multi-echo SE sequences) was also included in the EPG T_2_ fitting (Santini et al., 2021).

The T_1_ relaxation time in our study aligns with previously reported values (Han et al., 2003; Gold et al., 2004). However, as mentioned, no differences in T_1_ relaxation time were found between the three groups. The control group showed a slightly (although not significantly) shorter T_1_ value, potentially due to a higher fat fraction, since fat has a notably shorter T_1_ relaxation time compared to muscle. Unlike EPG-based T_2_ fitting, the VFA-based quantification method retrieves only a global T_1_ value (without being able to distinguish signal contribution from muscle water and fat).

Analysis of the IVIM parameters showed that both athlete cohorts tended to have higher perfusion fraction *f* values, consistent with previous results in leg muscles (Okamoto et al., 2012) and suggesting increased capillary density and perfusion as a long-term effect associated with training. Notably, the perfusion fraction in the strength cohort appears to be consistently higher, which may be a consequence of the targeted repeated exercises that powerlifters perform with the lumbar back muscle. Interestingly, the endurance cohort also shows a higher value of f (when compared to the control group). Although this result is not significant, it suggests a possible effect of non-targeted training on perfusion and appears to agree with Hoier and Hellsten (2014).

The two diffusion coefficients paint a more nuanced picture of different effects in the various cohorts and muscles. Specifically, while $D_{xy}$ shows an increasing trend that is most pronounced in the strength-training group, $D_{z}$ shows opposite signs in the two athlete cohorts. The axial diffusion coefficient ( $D_{z}$ ) is consistently higher than the radial diffusion coefficient, which aligns with the expected natural orientation of muscle fibers (Damon et al., 2002). In general, DWI is sensitive to microstructural alterations and adaptations in muscle tissue (Tan et al., 2022), although the full extent of how various muscle features affect DWI is not yet fully understood. Flück et al. (2019) performed a thorough analysis of very similar cohorts, but focused on biopsies of one muscle group in the thigh (*vastus lateralis*). They reported a higher myofibrillar volume density and a larger cross-sectional area of muscle fibers in their powerlifter cohort. Monte-Carlo simulations have revealed that muscle fiber diameter has the greatest influence on diffusion anisotropy and mean diffusivity (Berry et al., 2018). Thus, it could be speculated that the muscle hypertrophy observed by Flück et al. (2019) in the strength-training cohort is due to larger muscle fiber diameters, contributing to the higher value of radial diffusion $D_{xy}$ compared to the control group and the endurance cohort. Similarly, the lower axial diffusion coefficient $D_{z}$ in the strength cohort could be attributed to a higher macromolecular fraction (also due to hypertrophy), a factor not present in the endurance cohort (Mazzoli et al., 2021).

Measurements on capillary phantoms have linked the pseudo-diffusion coefficient $D^{*}$ to blood velocity (Cho et al., 2012; Schneider et al., 2019), and non-MRI-based studies have shown that the effect of training leads to an increase in vascular velocity and blood flow in both endurance and powerlifting athlete cohorts (Green et al., 2012; Welsch et al., 2013). Thus, it could be speculated that this is reflected in the significantly higher values for *D* ^*^ in the athlete cohorts of this study. In the Supplementary Material (section 1.1, Figure 1), a further split of the pseudo-diffusion coefficient $D^{*}$ along the axial and radial directions is shown, with a higher ${D_{z}}^{*}$ than ${D_{xy}}^{*}$ , consistent with (Haas and Nwadozi, 2015) who showed that capillaries are predominantly oriented along the direction of the muscle fiber. Finally, no significant correlations were found between the all the investigated parameters (apart from the expected positive correlation between *f* and *D* ^*^).

### 4.1 Limitations

Our study has several limitations: Only male subjects were analyzed, and the size of each cohort was relatively small, which impairs statistical power. Furthermore, the subjects were all measured at the same time of the day and had to refrain from training for 48 h before the measurements. While several different quantitative MRI studies in muscles at rest have used different rest periods before scanning, ranging from 6 h (Marth et al., 2022) to 24 h (Sun et al., 2018), the 48-hour rest interval chosen for this protocol was the most conservative approach that was still feasible given the athletes’ demanding training schedules. It is worth noting that although two studies (Maeo et al., 2017; Lyu et al., 2021), including one involving animal models, suggest that the effect of strenuous exercise could influence qMRI parameters for up to 72 h post-exercise, such a long rest period was not feasible for the athlete cohorts involved in our study.

The diffusion gradient scheme in our DWI acquisition was insufficient for full diffusion tensor reconstruction, necessitating the use of diffusion coefficients *D*_*z*_ and *D*_*xy*_. The use of these coefficients to approximate the axial and radial diffusivities relied on assumptions about the orientation of the muscle fibers, which meant that fiber dispersion could influence the results. While we do not have the data to quantify such influence, it may be noted that this assumption may be more accurate in the case of erector spinae compared to multifidus due to the differences in fiber orientation of each muscle. Therefore it is recommended that future studies include additional diffusion directions to enable the reconstruction of the full diffusion tensor, thus allowing for a more accurate analysis.

All regions of interest were drawn by hand, with the aim of excluding muscle-fat replacements, which are commonly present in the back muscles. Nevertheless, partial volume effects may have affected some voxels, especially in DWI images due to the low spatial resolution in the inferior-superior (IS) direction.

Finally, while training may primarily explain the cohort differences observed by qMRI in this study, other important factors such as diet/supplement intake, which is particularly prevalent among young athletes (Farup et al., 2014; Jovanov et al., 2019), were not considered in this work. More comprehensive research is needed to clearly differentiate between these two factors.

## 5 Conclusion and outlook

This study focused on the development of a comprehensive protocol based on a variety of parameters, including water T_2_, fat fraction, T_1_ and IVIM-based diffusion parameters. Application of this protocol to a group of athletes revealed significant differences in muscle water T_2_ and fat fraction which we believe is a valuable contribution to the growing (and at times conflicting) qMRI results associated with long-term effects of exercise in skeletal muscles. Furthermore, the reported differences in IVIM parameters may indicate long-term effects associated with different types of training, potentially reflecting underlying microstructural changes. However, it is crucial for future implementations of the DWI protocol to include additional directions to effectively disentangle the influence of muscle fiber direction and dispersion, which was not achievable with the current protocol. Given that T_1_ mapping, carefully corrected for possible B_1_ contamination, appears to provide little insight into the effects of training, it may be interesting for future research to abandon the VFA approach used in this protocol and employ more advanced methods, such as the recently proposed MR-fingerprinting water T_1_ method (Marty and Carlier, 2020), which could help disentangle the different signal contributions or highlight potential differences between the cohorts, or more advanced diffusion models of microstructure (e.g., NODDI or HARDI, as shown in the preliminary results of Adluru et al. (2017)). Alternatively, future research efforts could focus on correlating the investigated parameters with functional parameters (such as EMG) or exploring additional parameters such as T_1ρ_, which are significantly influenced by macromolecular concentration and may be related to intervertebral disc degeneration, which is also implicated in non-specific low back pain. Finally, the ability of the proposed protocol to capture adaptations at rest may offer new perspectives in the evaluation of changes in skeletal muscle associated with training and may aid in the treatment monitoring of nonspecific low back pain patients who cannot undergo examinations after a strenuous exercise session.

## Data Availability

The datasets presented in this article are not readily available because they are available upon reasonable request to the corresponding author. Requests to access the datasets should be directed to marta.maggioni@uni-jena.de.

## Appendix A1

DIPY, an open-source python-based project aimed at the analysis and visualization of DWI data (https://dipy.org/index.html) implements a version of a variable projection (VarPro) (Golub and Pereyra, 1973) approach by treating the signal fitting in each voxel as a sequence of three optimization problems: first, the global optimum with respect to *D* ^***^ and *D*_*x,y,z*_ is sought using a differential evolution algorithm (non-convex) and then used in a convex linear regression fitting of *S*_*0*_ and *f*; the obtained values are then refined jointly using non-linear least squares (Farooq et al., 2016; Fadnavis et al., 2019). Note that this approach processes each voxel independently, without leveraging spatial regularization. Additionally, all acquired volumes were used at every step, without splitting b-values.
