# Supplementary material for: Assessment of training-associated changes of the lumbar back muscle using a multiparametric MRI protocol
Source: Front Physiol. 2024 Oct 17;15:1408244. doi: 10.3389/fphys.2024.1408244 (PMC11524875; doi:10.3389/fphys.2024.1408244)
Supplement: Supplementary file 1 [file Table1.docx]

Supplementary Material

## Radial and axial pseudo-diffusion coefficients


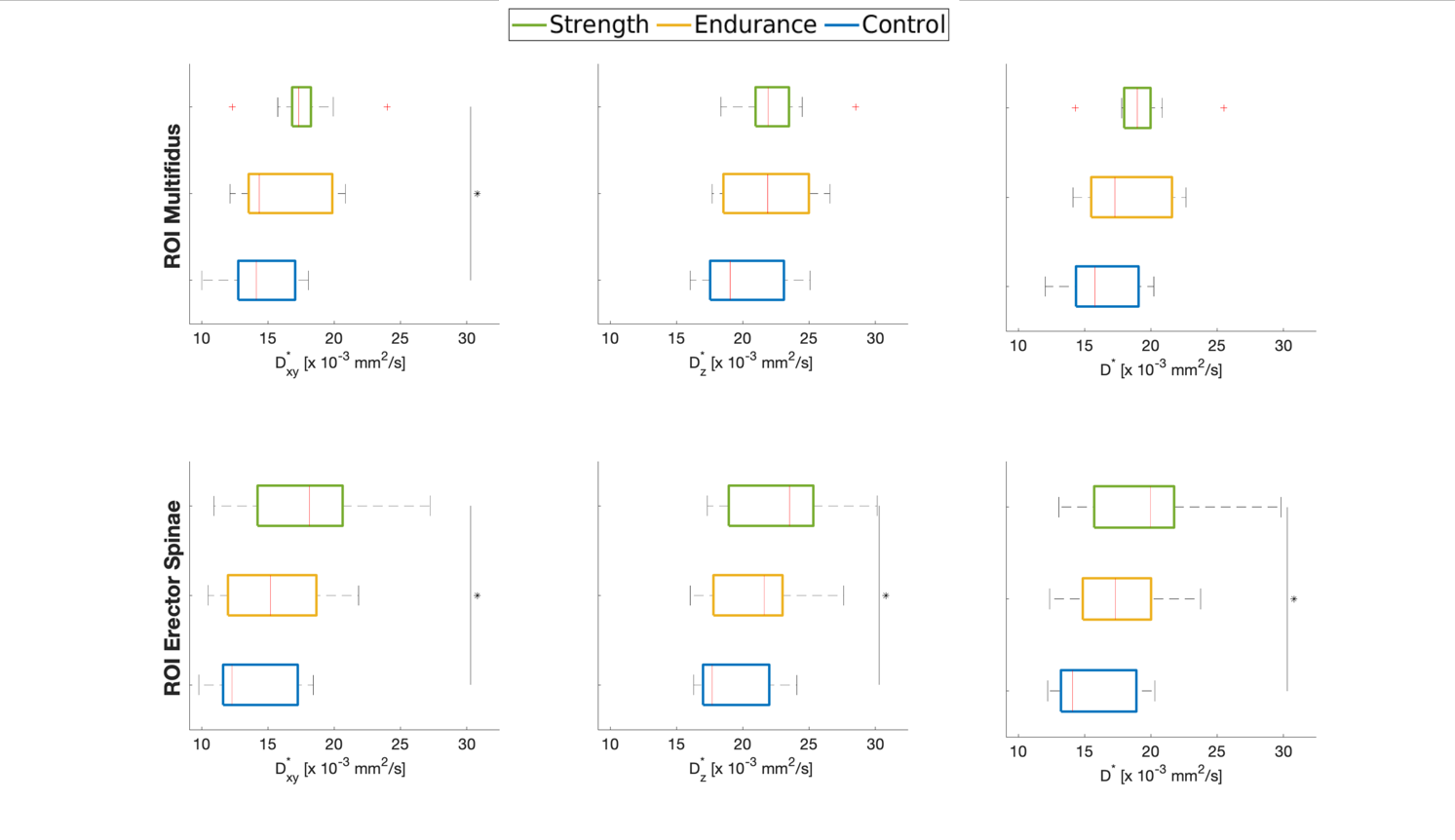


**Figure 1**. Boxplots of ROI-averaged D_xy_*, D_z_* and comparison with global D* in the multifidus (top row) and erector spinae (bottom row) muscles of the three cohorts analyzed in this work. * indicates the p-values, where significant, between indicated cohorts.

## Dixon measurements

**Table 1a**. Parameters of the 2-point Dixon clinical acquisition. TR stands for repetition time, TE for echo time and TA for acquisition time. VIBE stands for Volumetric interpolated breath-hold examination.

|  | VIBE Dixon |
| --- | --- |
| TR [ms] | 6.1 |
| TE [ms] | 2.46, 3.69 |
| Voxel [mm^3^] | 1.5$\times$1.5×3 |
| Averages | - |
| Slices | 40 |
| TA | 2 min |

**Table 2a**. Values of fat fraction calculated from the Dixon approach. *MF — M. multifidus; ES — M. erector spinae.*

| Cohort | Fat fraction  Dixon  (%)  MF | Fat fraction  Dixon  (%)  ES |
| --- | --- | --- |
| Control | 5 ± 3 | 3 ± 2 |
| Endurance | 0.7 ± 0.3 | 1.1 ± 0.2 |
| Strength | 1.05 ± 0.03 | 1.03 ± 0.02 |

- Fat fraction (Dixon) results for multifidus muscle

Kruskal-Wallis p-value=8 e-10

|  | Control | Endurance | Strength |
| --- | --- | --- | --- |
| Control | 1 | 4.4e-10 | 6e-6 |
| Endurance | 4.4e-10 | 1 | 0.45 |
| Strength | 6e-6 | 0.45 | 1 |

- Fat fraction (Dixon) for erector spinae muscle

Kruskal-Wallis p-value=1.5 e-06

|  | Control | Endurance | Strength |
| --- | --- | --- | --- |
| Control | 1 | 1.1e-04 | 5e-06 |
| Endurance | 1.1e-04 | 1 | 0.99 |
| Strength | 5e-06 | 0.99 | 1 |

## Illustration of all the muscles


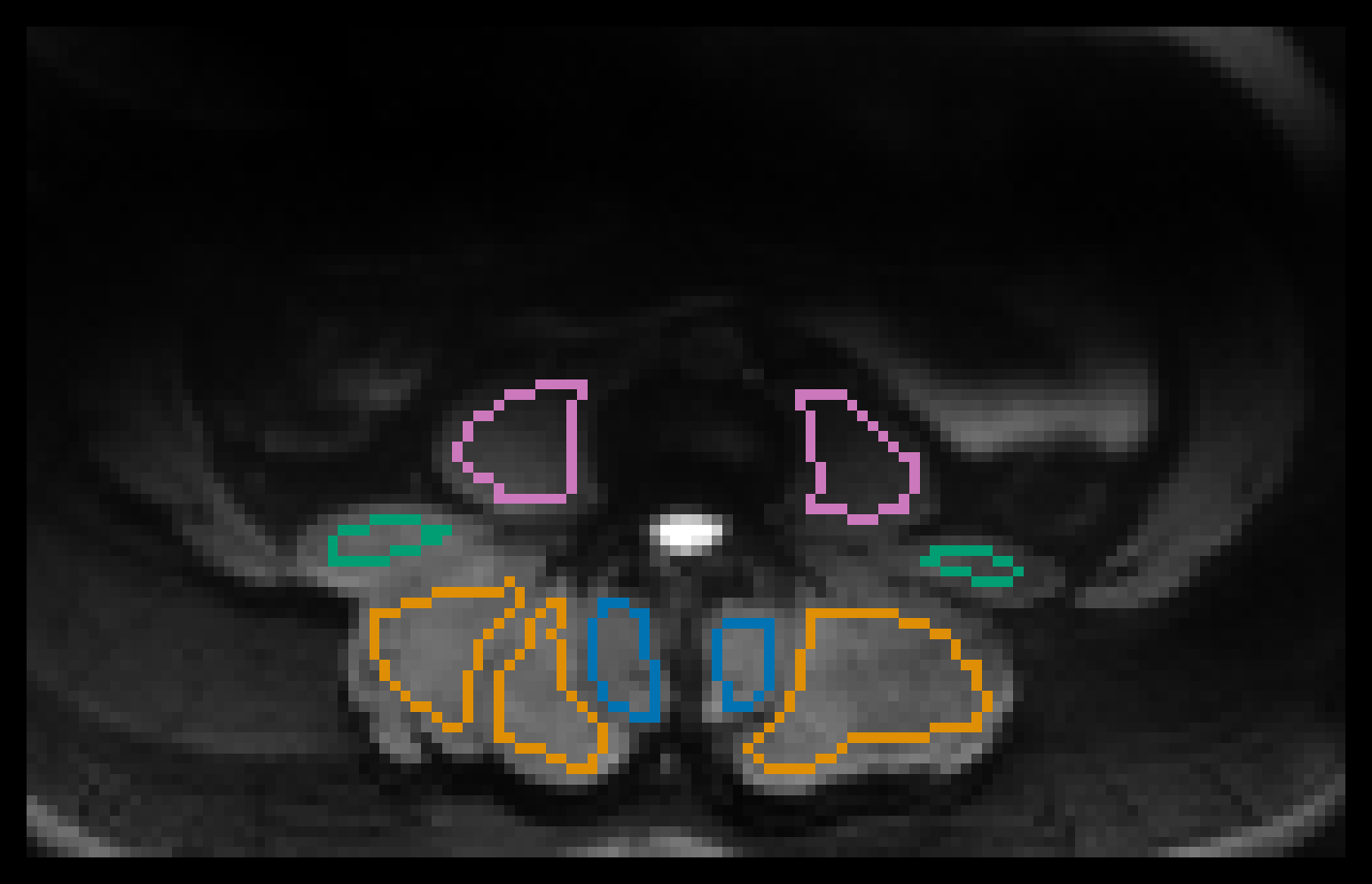


**Figure 2**.DWI signal (b=0) for a representative subject of the strength cohort. Note the significant drop of signal in the psoas major muscles (color coded in pink), tangentially affecting the quadratus lumborum, (color coded in green). Multifidus is indicated in blue and erector spinae in orange as in the manuscript text.

Additionally, the small number of voxels associated with the segmentation of the quadratus lumborum complicates consistent evaluation of this muscle across different cohorts.

## Representative maps for one subject of the strength and control group

**Figure 3**. Below example parameter maps for one strength and control group subject, respectively are shown. The grouping of the maps, indicated by the frame color, represents the acquisition from which they originate (VFA, TSE, or IVIM). Maps sharing a color bar are windowed identically. In the corrected T_1_ relaxation time map, the successful B_1_-field inhomogeneity correction is visible compared to the uncorrected map (*a*). Section (*b*) shows the water T_2_ and fat fraction maps extracted from the EPG. Section (*c*) shows the maps of the diffusion coefficients $D_{xy}$, *D_z_*, the pseudo-diffusion coefficient *D^*^*, and the perfusion fraction *f*. Additionally, the ROIs used for quantitative analysis are outlined on top of the map of *S_0_* (multifidus in blue and erector spinae in orange).


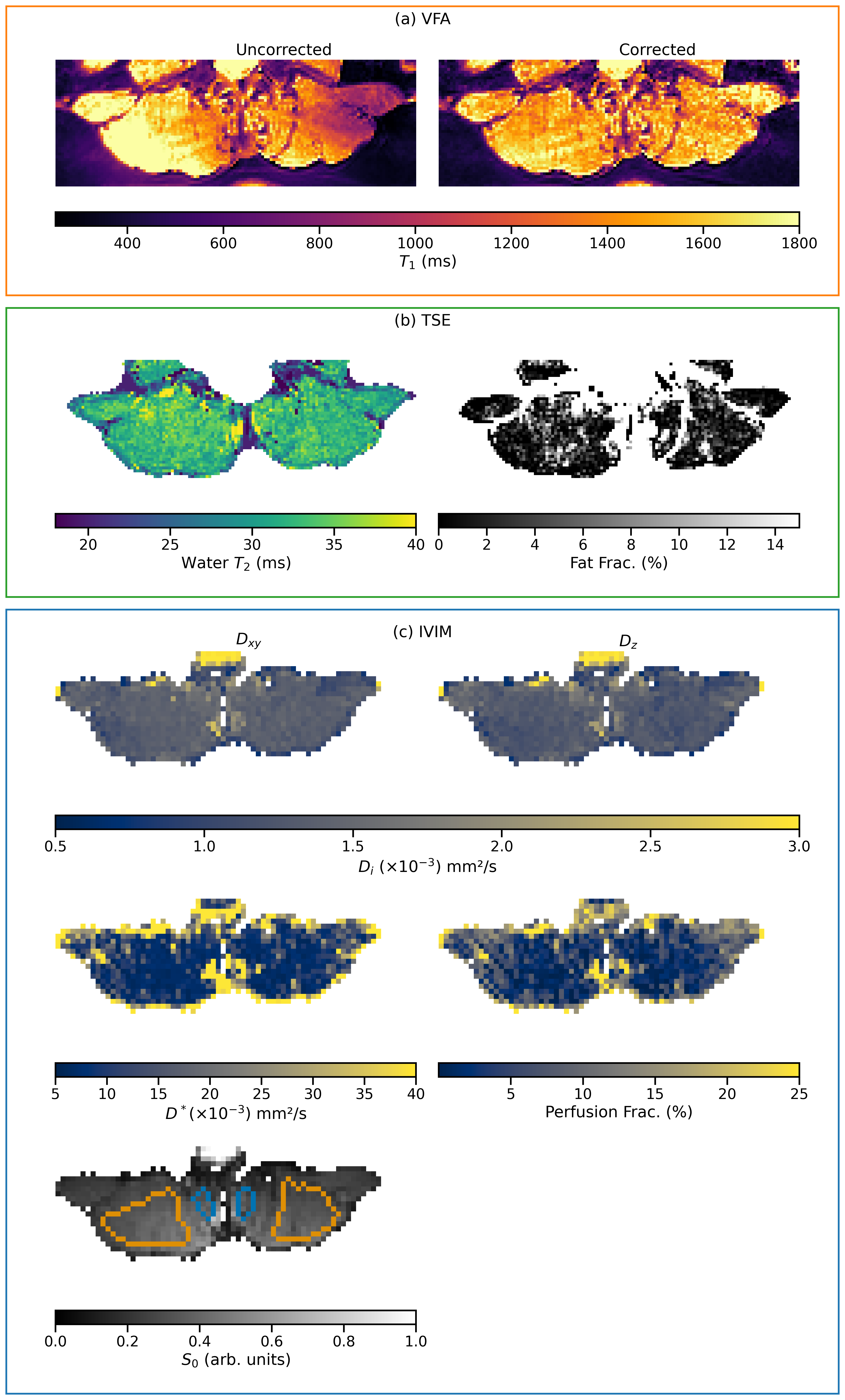

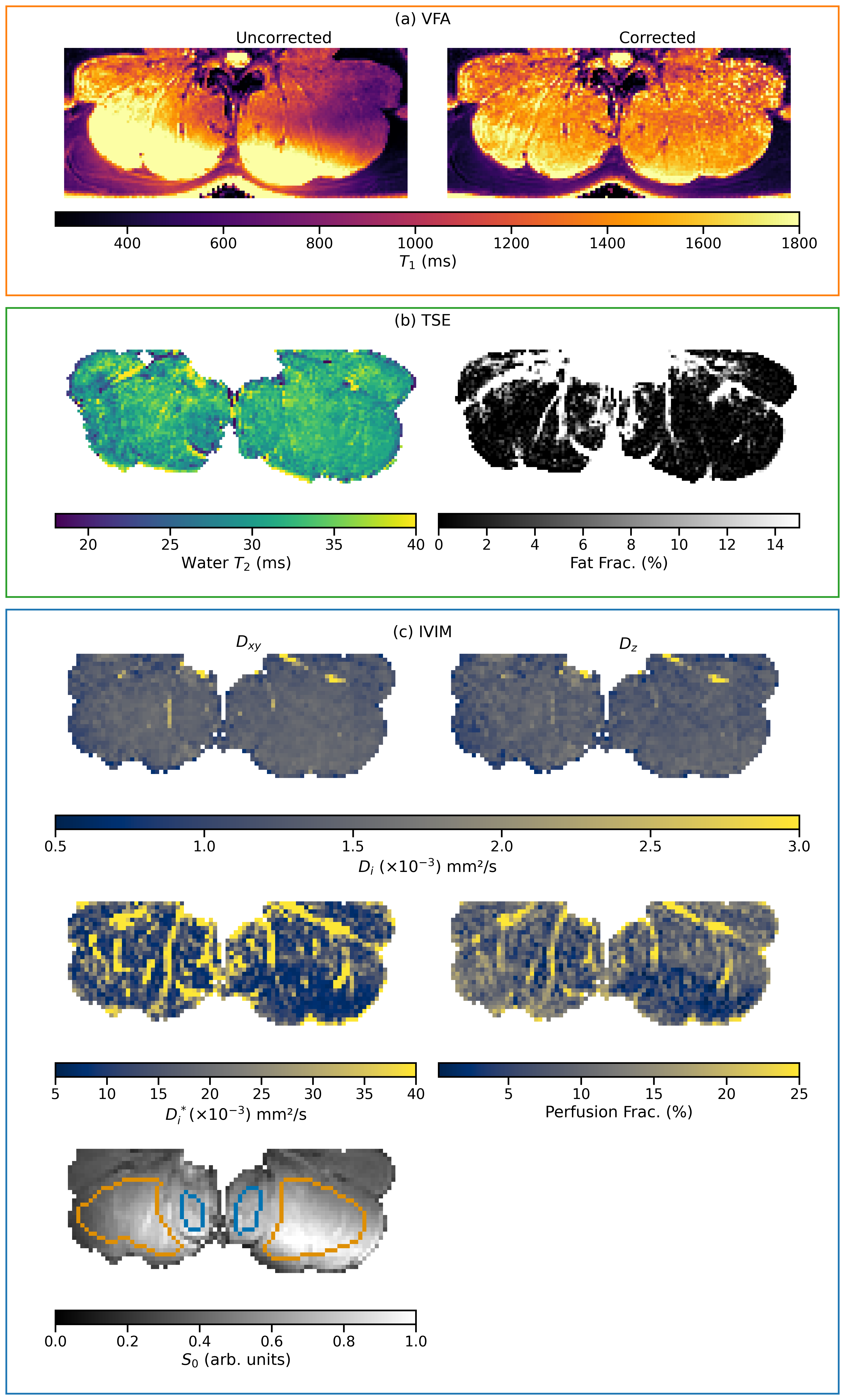


## Results from Kruskal-Wallis and Dunn’s post hoc comparison

- T_2_ results for multifidus muscle

Kruskal-Wallis p-value=0.020

|  | Control | Endurance | Strength |
| --- | --- | --- | --- |
| Control | 1 | 0.012 | 0.025 |
| Endurance | 0.012 | 1 | 0.845 |
| Strength | 0.025 | 0.845 | 1 |

- T_2_ results for erector spinae muscle

Kruskal-Wallis p-value=0.021

|  | Control | Endurance | Strength |
| --- | --- | --- | --- |
| Control | 1 | 0.018 | 0.017 |
| Endurance | 0.018 | 1 | 0.929 |
| Strength | 0.017 | 0.929 | 1 |

- Fat fraction results for multifidus muscle

Kruskal-Wallis p-value=2.5 e-07

|  | Control | Endurance | Strength |
| --- | --- | --- | --- |
| Control | 1 | 1.8e-04 | 1.1e-07 |
| Endurance | 1.8e-04 | 1 | 0.813 |
| Strength | 1.1e-07 | 0.813 | 1 |

- Fat fraction for erector spinae muscle

Kruskal-Wallis p-value=2.2 e-07

|  | Control | Endurance | Strength |
| --- | --- | --- | --- |
| Control | 1 | 3e-06 | 2e-06 |
| Endurance | 3e-06 | 1 | 0.986 |
| Strength | 2e-06 | 0.986 | 1 |

- T_1_ results for multifidus muscle

Kruskal-Wallis p-value=0. 290

- T_1_ results for erector spinae muscle

Kruskal-Wallis p-value= 0.796

- Perfusion fraction results for multifidus muscle

Kruskal-Wallis p-value=0.284

- Perfusion fraction results for erector spinae muscle

Kruskal-Wallis p-value=0.088

- Radial diffusion results for multifidus muscle

Kruskal-Wallis p-value=0.049

|  | Control | Endurance | Strength |
| --- | --- | --- | --- |
| Control | 1 | 0.063 | 0.001 |
| Endurance | 0.063 | 1 | 0.100 |
| Strength | 0.001 | 0.100 | 1 |

- Radial diffusion results for erector spinae muscle

Kruskal-Wallis p-value=0.025

|  | Control | Endurance | Strength |
| --- | --- | --- | --- |
| Control | 1 | 0.710 | 0.011 |
| Endurance | 0.710 | 1 | 0.058 |
| Strength | 0.011 | 0.058 | 1 |

- Axial diffusion results for multifidus muscle

Kruskal-Wallis p-value=0.047

|  | Control | Endurance | Strength |
| --- | --- | --- | --- |
| Control | 1 | 0.024 | 0.100 |
| Endurance | 0.024 | 1 | 0.001 |
| Strength | 0.100 | 0.001 | 1 |

- Axial diffusion results for erector spinae muscle

Kruskal-Wallis p-value=0.045

|  | Control | Endurance | Strength |
| --- | --- | --- | --- |
| Control | 1 | 0.044 | 0.381 |
| Endurance | 0.044 | 1 | 0.026 |
| Strength | 0.381 | 0.026 | 1 |

- Pseudo-diffusion results for multifidus muscle

Kruskal-Wallis p-value=0.136

- Pseudo-diffusion results for erector spinae muscle

Kruskal-Wallis p-value=0.050

|  | Control | Endurance | Strength |
| --- | --- | --- | --- |
| Control | 1 | 0.201 | 0.020 |
| Endurance | 0.201 | 1 | 0.323 |
| Strength | 0.020 | 0.323 | 1 |

- Age

Kruskal-Wallis p-value=0.097

- BMI

Kruskal-Wallis p-value=0.001

|  | Control | Endurance | Strength |
| --- | --- | --- | --- |
| Control | 1 | 0.0535 | 0.0044 |
| Endurance | 0.0535 | 1 | 0.0002 |
| Strength | 0.0044 | 0.0002 | 1 |
